# Preoperative Serum CA125 Levels Predict the Prognosis in Hyperbilirubinemia Patients With Resectable Pancreatic Ductal Adenocarcinoma

**DOI:** 10.1097/MD.0000000000000751

**Published:** 2015-05-21

**Authors:** Tao Chen, Min-Gui Zhang, Hua-Xiang Xu, Wen-Quan Wang, Liang Liu, Xian-Jun Yu

**Affiliations:** From the Pancreatic Cancer Institute (TC, M-GZ, H-XX, W-QW, LL, X-JY), Fudan University, and Department of Pancreas and Hepatobiliary Surgery, Fudan University Shanghai Cancer Center, Shanghai; and Department of Ophthalmology (M-GZ), Shanghai Tenth People's Hospital, Tongji University School of Medicine, Shanghai, China.

## Abstract

Serum carbohydrate antigen 19-9 (CA19-9) is widely used to predict the prognosis for pancreatic ductal adenocarcinoma (PDAC). However, hyperbilirubinemia and the CA19-9 nonsecretor phenotype restrict the usage of serum CA19-9 alone. The goal of this study was to confirm the prognostic role of preoperative serum CA125 in PDAC, especially in patients with jaundice.

A total of 211 patients with resected PDAC were eligible for this retrospective study, and were classified into 2 groups based on serum bilirubin levels. The prognostic significance of all clinicopathologic factors was evaluated by univariate and multivariate analyses, and the performance of each factor in predicting overall survival (OS) and recurrence-free survival (RFS) was compared.

High preoperative CA125, high TNM stage, and lymph node metastasis were independent risk predictors for OS and RFS in all patients and the 2 subgroups, but high CA19-9 was only significant when considering all patients and those with nonelevated bilirubin. Using time-dependent receiver-operating characteristic analysis, better predictive performance for OS and RFS was observed for serum CA19-9 as compared to serum CA125 in these patients.

High serum CA125 can independently predict poor prognosis. Importantly, in PDAC patients with hyperbilirubinemia, preoperative serum CA125 can predict the prognosis, whereas CA19-9 cannot. Preoperative CA19-9 had better predictive performance for survival than CA125, and the performance of CA19-9 did not decline between all patients and those with nonelevated bilirubin, but was significantly affected by hyperbilirubinemia.

## INTRODUCTION

Pancreatic ductal adenocarcinoma (PDAC) is one of the most lethal solid organ malignancies, with a 5-year survival rate of 5%. Pancreatectomy offers the only potential for cure, but is only possible in a minority of patients, and the 5-year survival rate after resection is <20%.^1^ The high mortality rate is mainly due to the tendency of PDAC cells to metastasize early. Thus, we need to identify specific early detection indicator(s) to adjust surgical and perioperative therapy in potentially resectable patients.

Many PDAC tumor-associated antigens have been studied, including serum carbohydrate antigen 19-9 (CA19-9), CA125, and carcinoembryonic antigen (CEA).^2,3^ Serum CA19-9 is used as a diagnostic adjunct and a prognostic marker, and an elevated preoperative CA19-9 level is associated with poor prognosis.^4,5^ However, approximately 5% to 14% of the population is Lewis antigen A and B negative (Le^a−b−^), and is also considered CA19-9 nonsecretory (CA19-9 < 5 U/mL),^6^ which is correlated with poor survival.^7^ Approximately 67.5% of patients with tumors located at the head of the pancreas have hyperbilirubinemia,^8^ and obstructive jaundice induces an increase in serum CA19-9 levels.^8,9^ Hence, preoperative measurement of CA19-9 levels in serum should be performed after biliary decompression is complete, but resection should not be delayed to wait for bilirubin level normalization. Because of obstructive jaundice and the CA19-9 nonsecretor phenotype, baseline preoperative serum CA19-9 levels cannot be used alone in routine clinical practice.^2,10^ As there is no preoperative serum prognostic marker for obstructive jaundice patients, patients with hyperbilirubinemia are typically excluded from their previous studies.^4,6,9–11^ Therefore, there is an urgent need to identify specific biomarkers to better stratify patients for initial treatment and evaluate the response to treatment.

CA125 is a high-molecular-weight mucin-like glycoprotein that is overexpressed on the surface of ovarian cancer cells and is secreted by other cancer cells types.^4,11,12^ It is described as the most useful serologic marker for ovarian cancer detection and management,^8,13^ and plays a potential role in PDAC pathogenesis and diagnosis.^3,8,14^ Our previous studies demonstrated that as an independent predictor, serum CA125 levels were related to distant metastasis. Furthermore, a preoperative serum signature of CEA^+^/CA125^+^/CA19-9 ≥1000 U/mL could be a useful preoperative predictor for metastasis in PDAC patients.^15^ This signature could also be used for individualized treatment, especially in patients with borderline resectable PDAC. More importantly, serum CA125 levels may be independent of serum bilirubin levels.^8,16,17^ The purpose of the present investigation was to assess the prognostic impact of preoperative serum CA125 in PDCA patients who were undergoing curative surgical resection, especially in those with hyperbilirubinemia.

## PATIENTS AND METHODS

### Patient Selection and Data Collection

A total of 211 consecutive PDAC patients undergoing pancreatic resection were enrolled in this retrospective study between March 2010 and May 2012 at the Department of Pancreas and Hepatobiliary Surgery, Shanghai Cancer Center, Fudan University, Shanghai, China. The inclusion criteria were definitive PDAC diagnosis by pathology; no prior anticancer treatment before surgery or history of a second primary tumor; surgical resection with the cut surface being free of cancer by histologic examination; preoperative CA19-9 levels of >5 U/mL; and complete clinicopathologic and follow-up data after surgery.

Peripheral venous blood samples were taken from every patient within 14 days prior to surgery. Serum CA19-9 and CA125 levels were measured using a radioimmunoassay kit (Abbott-Diagnostics, Chicago, IL). Preoperative biliary drainage was performed by endoscopic retrograde cholangiopancreatography (ERCP) in 62 patients. In 4 patients with failed ERCP attempts, percutaneous transhepatic cholangiodrainage (PTCD) was used as a rescue option. The variables evaluated included age, sex, tumor size, location, differentiation status, lymph node involvement, TNM stage, neural invasion, and microvascular invasion. In general, patients with positive lymph nodes or/and metastases after resection received standard gemcitabine-based chemotherapy or chemoradiation therapy with 5-FU followed by gemcitabine. The recommended upper limit for normal CA19-9 is 37 U/mL.^11,18^ X-tile plots were created by dividing serum CA125 into two populations: low and high. All possible divisions of serum CA125 were assessed by the log-rank test for survival, and the best cutoff value for serum CA125 was obtained.^19^

The study was approved by the Shanghai Cancer Center Research Ethics Committee. Informed consent was obtained from each patient according to the committee's regulations.

### Follow-Up

A computed tomography (CT) scan of the abdomen was done every 3.0 months during the first year. Postoperative patients had a median follow-up of 20.4 months (range 9.3 – 65.7) and were followed-up every 3 months until they die. Conventionally, the detection of recurrence is dependent on several noninvasive inspections, including ultrasound, CT, magnetic resonance imaging (MRI), and positron emission tomography (PET)/CT. If recurrence was suspected, a CT or MRI scan was done immediately. Overall survival (OS) was defined as the interval between the dates of surgery and death, or surgery and the last follow-up. Recurrence-free survival (RFS) was defined as the interval between the dates of surgery and tumor recurrence, or surgery and the last follow-up.

### Statistical Analysis

All statistical computations were performed using the Statistical Package for Social Science (SPSS, Inc., Chicago, IL) software, version 16.0 for Windows. For statistical analyses, the descriptive data were expressed as mean ± standard deviation. The Student *t* test or Pearson correlation test was applied to analyze quantitative variables. OS and RFS were displayed using Kaplan–Meier survival curves with 95% confidence intervals (CIs) and the differences between subgroups were compared using the log-rank test. All risk factors identified by univariate analysis were adopted in multivariate Cox proportional hazard analysis. A time-dependent receiver-operating characteristic (ROC) curve was applied to evaluate the predictive performance of the abovementioned parameters, and the integrated area under the curve (IAUC) was calculated using R statistical packages (“survival ROC” and “survcomp”; www.r-project.org). The comparisons were done using the 2-tailed log-rank test. A probability *P* value of ≤0.05 was considered statistically significant.

## RESULTS

### Demographic and Clinical Characteristics

In this retrospective study, 12 patients had preoperative CA19-9 levels of <5 U/mL (5 patients received neoadjuvant chemotherapy); 126 patients had a bilirubin of <2 mg/dL, and 73 patients had hyperbilirubinemia at the time the CA19-9 was measured. Of the 199 patients, 124 (62.3%) patients underwent pancreaticoduodenectomy, 72 (36.2%) underwent distal pancreatectomy, and 3 (1.5%) underwent total pancreatectomy. The median baseline preoperative serum levels of CA19-9 and CA125 were 179.20 (range, 5.90–2111.00) U/mL and 17.48 (range, 2.57–251.50) U/mL, respectively. In a paired analysis, the median baseline preoperative serum levels of CA19-9 and CA125 were 145.60 (range, 5.90–2111.00) U/mL and 16.30 (range, 2.57–251.5) U/mL, respectively (Table 1). Postoperative adjuvant chemotherapy was administered to 169 of 199 patients. Of the 126 patients without elevated bilirubin, there were 91 patients with elevated preoperative serum CA19-9 levels (>37 U/mL). In the remaining 73 patients with hyperbilirubinemia, 66 had elevated serum CA19-9 levels and 37 had elevated serum CA125 levels. The majority of patients with hyperbilirubinemia underwent PBD by ERCP or PTCD (90.4%) (6 days, range, 4–16 days). Demographic and clinical characteristics were summarized in Table 1.

**TABLE 1:**
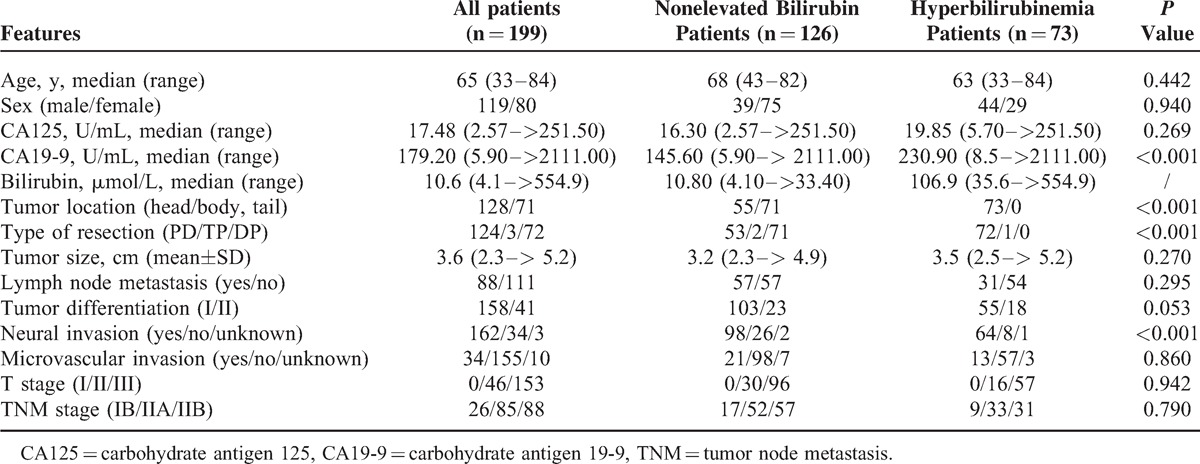
Clinicopathological Features of Patients

**FIGURE 1:**
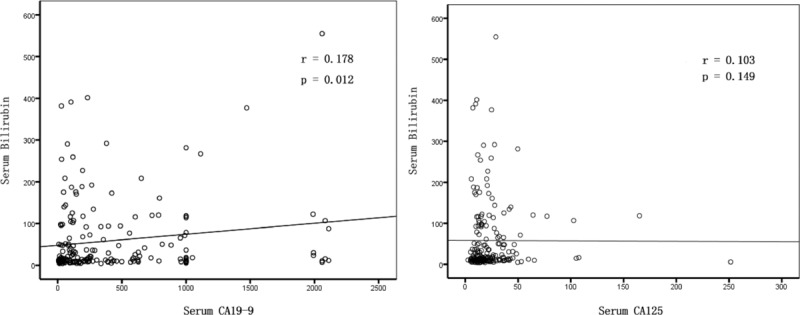
Correlation between bilirubin and serum CA19-9 levels (A) and serum CA125 levels (B). Preoperative serum bilirubin levels were correlated with serum CA19-9 (*r* = 0.178, *P* = 0.012), but were not correlated with serum CA125 (*r* = 0.103, *P* = 0.149). CA125 = carbohydrate antigen 125, CA19-9 = carbohydrate antigen 19-9.

In all patients, a positive correlation was observed between serum CA19-9 and bilirubin (*r* = 0.180, *P* = 0.012), but not CA125 (*r* = 0.103, *P* = 0.149) (Figure 1). Neither sex nor age resulted in significantly different preoperative CA19-9 (*P* = 0.686, *P* = 0.233, respectively) or CA125 (*P* = 0.836, *P* = 0.998, respectively). Serum CA125 was associated with serum CA19-9 (*P* = 0.005), lymph node metastasis (*P* = 0.009), and TNM stage (*P* = 0.008). In patients without elevated bilirubin, serum CA19-9 was associated with lymph node metastasis (*P* < 0.001) and TNM stage (*P* < 0.001). There was not a significant correlation between CA19-9 or CA125 and other clinicopathological characteristics, including tumor location, differentiation, microvascular invasion, neural invasion, and tumor size. In patients with hyperbilirubinemia, the correlation between CA19-9 and bilirubin was marginal with a very low correlation coefficient (*r* = 0.227, *P* < 0.001). Serum CA125 levels had a statistically significant correlation with lymph node metastasis (*P* = 0.008) and TNM stage (*P* = 0.001) (Table 2).

**TABLE 2:**
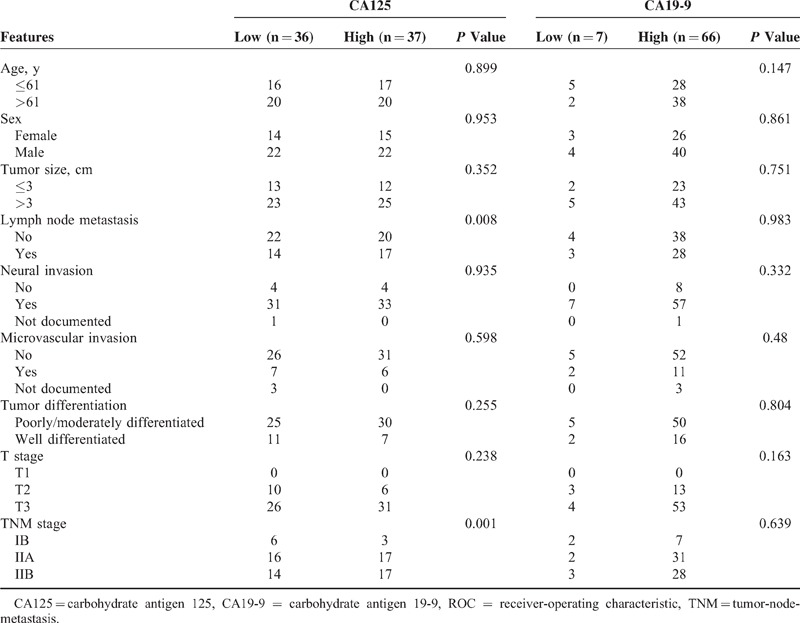
Relations Between the Parameters and Pathological Features in Hyperbilirubinemia Patients

### Survival in Hyperbilirubinemia and Nonelevated Bilirubin Groups

At the end of this study, 83 patients (41.7%) were still alive, and 136 patients (68.3%) had disease recurrence. Overall survival rates of the 199 patients at 1, 2, and 3 years were 91.5%, 61.4%, and 38.0%, respectively. The RFS rates at 1, 2, and 3 years were 52.8%, 27.4%, and 22.0%, respectively. The median times of OS and RFS were 20.3 (95% CI: 17.0–23.6) and 12.6 (95% CI: 10.3–14.9) months. Seventy-four patients (59.2%) died and 84 patients (66.7%) had tumor recurrence in the subgroup of 126 patients with hyperbilirubinemia, and 42 patients died (57.5%) and 52 patients (71.2%) had tumor recurrence in the subgroup of 73 patients with the nonelevated bilirubin. PBD was not correlated with OS (*P* = 0.215) or RFS (*P* = 0.142). The Kaplan–Meier curves relating serum bilirubin to OS (*P* = 0.314) and RFS (*P* = 0.276) are shown in Table 3. Median survival time in the hyperbilirubinemia group was 19.6 months (95% CI: 13.5–25.6) versus 22.7 months (95% CI: 18.7–26.7) in the nonelevated bilirubin group.

**TABLE 3:**
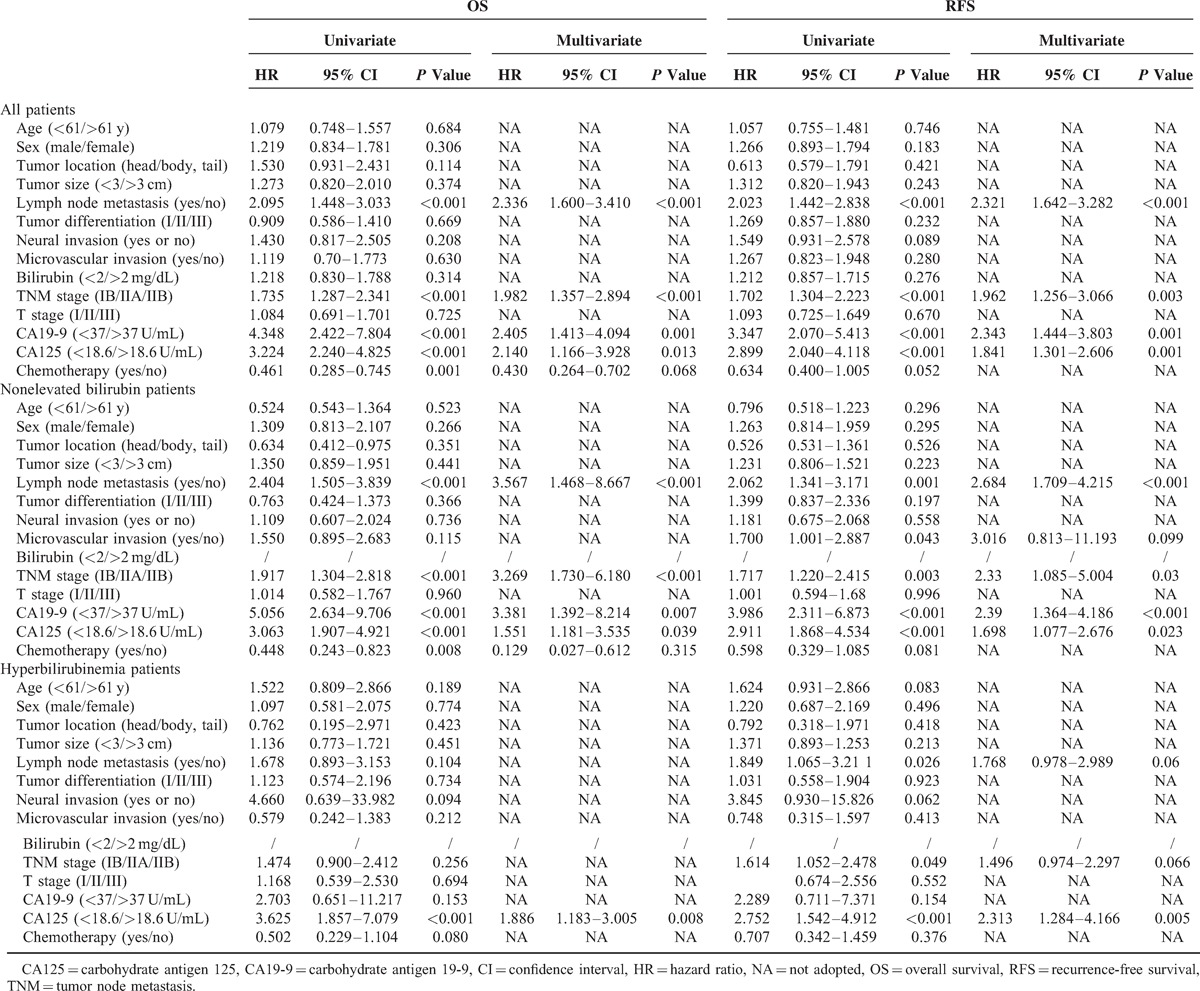
Univariate and Multivariate Analyses of Factors Associated With OS and RFS

### Serum CA125/CA19-9 Levels and Survival

**FIGURE 2:**
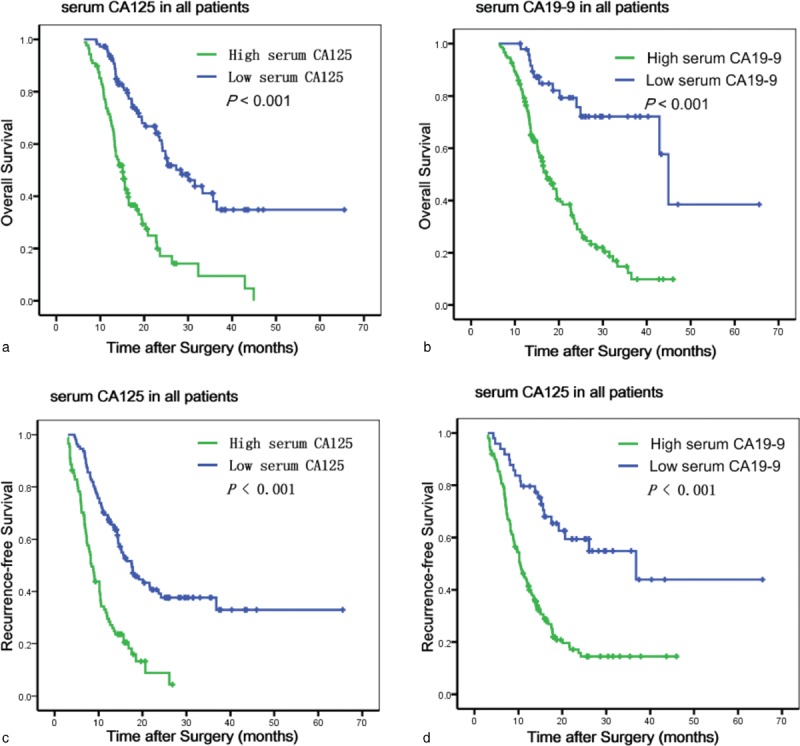
Kaplan–Meier survival curves for OS (a and b) and RFS (c and d) according to CA125 levels and CA19-9 levels in all patients. CA125 = carbohydrate antigen 125, CA19-9 = carbohydrate antigen 19-9, OS = overall survival, RFS = recurrence-free survival.

**FIGURE 3:**
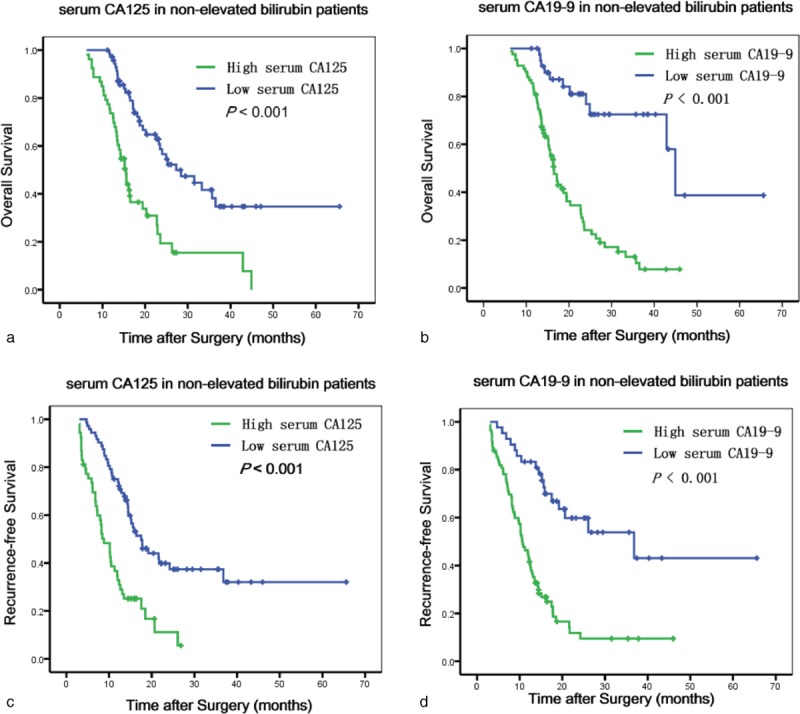
Kaplan–Meier survival curves for OS (a and b) and RFS (c and d) according to CA125 levels and CA19-9 levels in patients with nonelevated bilirubin. CA125 = carbohydrate antigen 125, CA19-9 = carbohydrate antigen 19-9, OS = overall survival, RFS = recurrence-free survival.

The best cutoff value for CA125 level was 18.6 U/mL, as calculated by X Tile. Univariate analyses indicated that elevated serum CA125 and CA19-9 were statistically significant risk predictors for poor OS and RFS (Figures 2 and 3) in overall and nonelevated bilirubin patients, in addition to high TNM stage, lymph node metastasis, and chemotherapy. In the multivariate analyses, serum CA125 (in all patients: *P* = 0.013, hazard ratio [HR] = 2.140, 95% CI: 1.166–3.928 for OS; *P* = 0.001, HR = 1.841, CI: 1.301–2.606 for RFS; in nonelevated patients: *P* = 0.039, HR = 1.551, 95% CI: 1.181–3.535 for OS; *P* = 0.023, HR = 1.698, 95% CI: 1.077–2.676 for RFS), CA19-9 (in all patients: *P* = 0.001, HR = 2.405, 95% CI: 1.413–4.094 for OS; *P* = 0.001, HR = 2.343, CI: 1.444–3.803 for RFS; in nonelevated patients: *P* = 0.007, HR = 3.381, 95% CI: 1.392–8.214 for OS; *P* < 0.001, HR = 2.390, 95% CI: 1.364–4.186 for RFS), high TNM stage, and lymph node metastasis that were analyzed as continuous or categorized variables were significantly associated with an increased risk of recurrence and death after adjusting for age, sex, tumor size, location, differentiation, neural invasion, and microvascular invasion.

**FIGURE 4:**
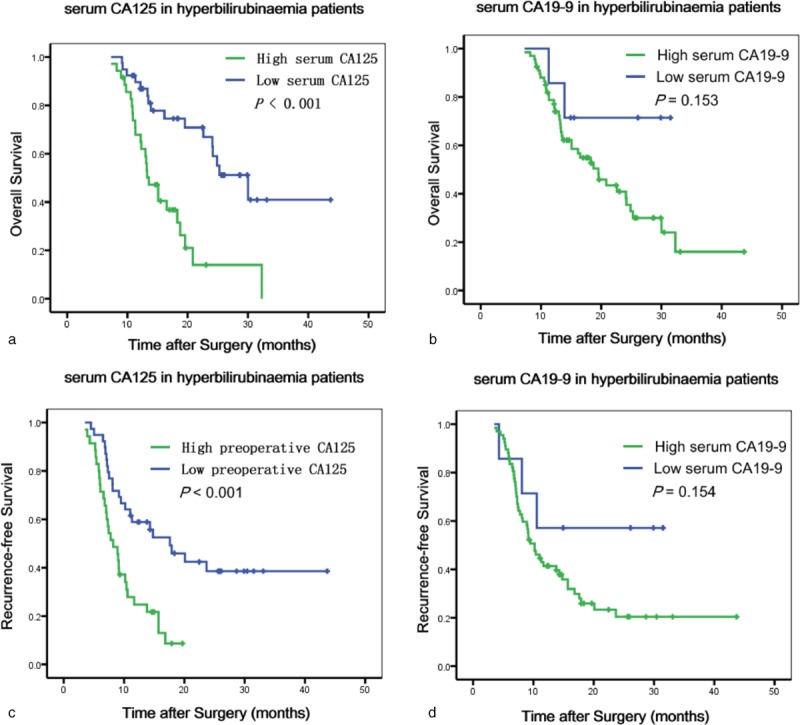
Kaplan–Meier survival curves for OS (a and b) and RFS (c and d) according to CA125 levels and CA19-9 in patients with hyperbilirubinemia. CA125 = carbohydrate antigen 125, CA19-9 = carbohydrate antigen 19-9, OS = overall survival, RFS = recurrence-free survival.

In hyperbilirubinemia patients, elevated serum CA125, but not CA19-9, was a statistically significant risk predictor for poor OS and RFS in the univariate analyses (Figure 4). Patients with normal CA125 had increased survival compared to those with elevated CA125 (median survival 20.1 vs 9.1 months, *P* < 0.001). Preoperative serum CA125 was an independent risk predictor for OS (*P* = 0.008, HR = 1.886, 95% CI: 1.183–3.005) and RFS (*P* = 0.005, HR = 2.313, 95% CI: 1.284–4.166) in the multivariate analyses. The values for the univariate and multivariate tests for every category considered are summarized in Table 3.

### Assessment of Predictive Performance

**FIGURE 5:**
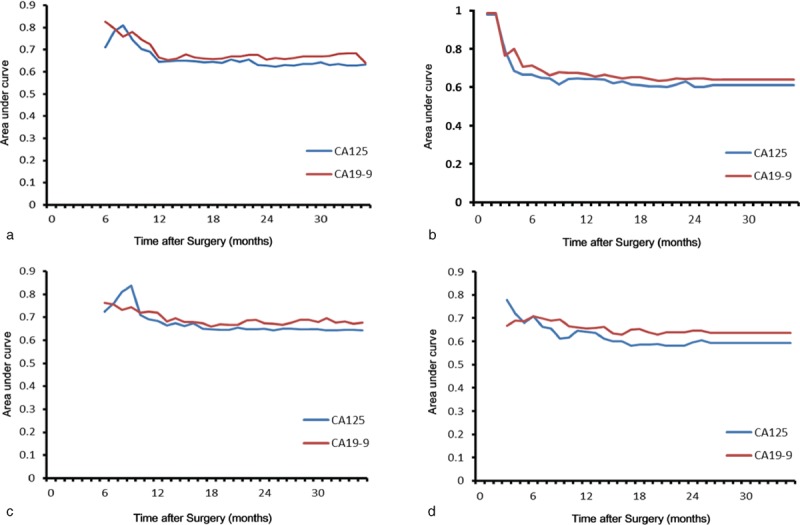
Time-dependent ROC curve analysis for predictions of OS (a and c) and RFS (b and d). Better predictive performances for both OS and RFS were achieved by CA19-9 levels compared to CA125 levels in all patients and those with nonelevated bilirubin. CA125 = carbohydrate antigen 125, CA19-9 = carbohydrate antigen 19-9, OS = overall survival, RFS = recurrence-free survival, ROC = receiver-operating characteristic.

All clinicopathologic factors identified by multivariate survival analyses were used for time-dependent ROC analyses. Better predictive performances for both OS and RFS were achieved by serum CA19-9 levels in all patients (CA125: IAUC = 0.660 for OS, IAUC = 0.650 for RFS; CA19-9: IAUC = 0.688 for OS, IAUC = 0.680 for RFS) and in nonelevated bilirubin patients (CA125: IAUC = 0.673 for OS, IAUC = 0.616 for RFS; CA19-9: IAUC = 0.692 for OS; IAUC = 0.653 for RFS) (Figure 5). The IAUCs for serum CA125 levels were 0.632 for OS and 0.622 for RFS in the patients with hyperbilirubinemia. The IAUCs for serum CA125 and CA19-9 levels between all patients and those with nonelevated bilirubin were not significantly different (CA125: *P* = 0.563 for OS, *P* = 0.673 for RFS; CA19-9: *P* = 0.463 for OS and *P* = 0.641 for RFS).

## DISCUSSION

Patients who have undergone surgical resection for PDAC have a dismal prognosis.^1^ For patients with potentially resectable disease, there is an urgent need to have a detection indicator(s) to adjust perioperative therapy and predict prognosis. Serum CA19-9 is the most widely used marker for the diagnosis, prognosis of pancreatic cancer, and is used to monitor postoperative recurrence and metastasis.^4,20–22^ In retrospective analyses of PDAC patient survival, elevated baseline preoperative elevated serum CA19-9 levels are indicative of poor prognosis.^4,9,10,23^ However, preoperative serum CA19-9 levels cannot be used for every PDAC patient. For example, some PDAC patients have hyperbilirubinemia, which induces an increase in serum CA19-9 levels^6^ that decrease once the jaundice is remedied. Approximately 67.5% of patients with a tumor in the head of the pancreas have hyperbilirubinemia.^8^ Moreover, CA19-9 nonsecretors have low serum CA19-9 levels, even if they have distant metastatic disease and those that underwent curative resection of their pancreatic cancer had a poor prognosis.^7^ Therefore, CA19-9 cannot be used in patients with increased bilirubin levels or CA19-9 nonsecretors. In this study, neither serum CA125 nor CA19-9 levels significantly impacted OS or RFS in the 12 nonsecretors evaluated, and they were excluded from this study. The prognostic value of CA19-9 was significantly affected by serum bilirubin in patients with hyperbilirubinemia. At this time, there is still no suitable preoperative serum indicator to predict prognosis in PDAC patients with hyperbilirubinemia.

Metabolic tumor burden (MTB), as measured by ^18^F-fluorodeoxyglucose (^18^F-FDG) PET/CT is an effective prognosticator for PDAC survival, and superior to preoperative serum CA19-9.^15^ However, because of its costliness, the application of ^18^F-FDG PET/CT examination is restricted in routine clinical practice. Therefore, simple and effective assays, such as tumor markers, are needed as supplemental tools to predict the prognosis for resectable PDAC. Our investigation identifies a potentially predictive role for preoperative serum CA125 in PDAC, especially in patients with obstructive jaundice.

CA125, which is encoded by the *MUC16* gene, is primarily known as a useful serological marker for the clinical management of ovarian cancer. In recent years, others have detected elevated expression of CA125 in the serum of PDAC patients, and have proposed a role for serum CA125 as a predictor of survival in these patients.^8,16,17,24^ More importantly, serum CA125 level is not affected by serum bilirubin.^16,25^ In this study, serum CA125 levels were had no correlation with serum bilirubin levels. In both univariate and multivariate analyses, elevated preoperative CA125 was an independent prognostic factor for OS and RFS in all PDAC patients, patients with nonelevated bilirubin and patients with hyperbilirubinemia. However, serum CA19-9 was not significantly associated with mortality after surgery for patients with jaundice. To our knowledge, this is the first study to demonstrate a prognostic role for serum CA125 in PDAC patients with hyperbilirubinemia.

Because surgery in jaundiced patients increases the risk for postoperative complications, preoperative biliary drainage (PBD) is often performed.^26^ There have been several studies investigating the effects of PBD on survival in patients undergoing pancreatoduodenectomy for pancreatic head cancer. PBD, as compared to surgery alone, had no effect on overall survival for those with pancreatic head cancer.^26–29^ In this study, the majority of patients with hyperbilirubinemia underwent PBD by ERCP or PTCD, and PBD was not correlated with OS or RFS. Therefore, PBD had little impact on the research results.

To evaluate the predictive performance of preoperative serum CA125 and CA19-9, we used time-dependent ROC curve analyses. In this retrospective study, preoperative serum CA19-9 levels had better predictive performances for predicting OS and RFS than serum CA125 levels in all patients and patients with nonelevated bilirubin. Interestingly, we found that the prognostic value of serum CA19-9 did not decline, when taking into account serum bilirubin (patients with nonelevated bilirubin and all patients). That is to say, serum bilirubin levels did not markedly affect serum CA19-9 levels in the entire group of PDAC patients who underwent surgical treatment. However, the prognostic value of CA19-9 was significantly affected by serum bilirubin in patients with hyperbilirubinemia.

Our study was inherently limited by its retrospective design. The patient sample size was not sufficient, especially in CA19-9 nonsecretor group. A portion of patients could not receive postoperative adjuvant therapy, which could impact survival time and lead to deviations in the analysis. Perioperative therapeutic delay resulting from hyperbilirubinemia was also a confounding variable in our study. Furthermore, serum CA125 is not a unique marker for PDAC, and can rise in response to other malignant and benign conditions, including cardiovascular disease, chronic liver disease, endometriosis, ovarian cancer, and other ovarian diseases.^12,30^

In conclusion, preoperative serum CA125 is an independent risk predictor that can predict the prognosis in PDAC patients with hyperbilirubinemia. Although hyperbilirubinemia markedly increased serum CA19-9 levels, the prognostic value of serum CA19-9 was not impacted in all patients and those with nonelevated bilirubin. Preoperative CA19-9 has a better predictive performance for OS and RFS than preoperative CA125 in these patients, and should be used for prognosis in patients with nonelevated bilirubin levels.
